# Complement C3c as a Biomarker in Heart Failure

**DOI:** 10.1155/2013/716902

**Published:** 2013-12-30

**Authors:** A. Frey, G. Ertl, C. E. Angermann, U. Hofmann, S. Störk, S. Frantz

**Affiliations:** ^1^Department of Internal Medicine I, University Hospital Würzburg, Oberdürrbacher Straße 6, 97080 Würzburg, Germany; ^2^Comprehensive Heart Failure Center, University of Würzburg, Straubmühlweg 2a, Haus A9, 97078 Würzburg, Germany

## Abstract

*Introduction.* Experimental data indicates an important role of the innate immune system in cardiac remodeling and heart failure (HF). Complement is a central effector pathway of the innate immune system. Animals lacking parts of the complement system are protected from adverse remodeling. Based on these data, we hypothesized that peripheral complement levels could be a good marker for adverse remodeling and prognosis in patients with HF. *Methods and Results.* Since complement activation converges on the complement factor C3, we measured serum C3c, a stable C3-conversion product, in 197 patients with stable systolic HF. Subgroups with normal and elevated C3c levels were compared. C3c levels were elevated in 17% of the cohort. Patients with elevated C3c levels exhibited a trend to better survival, slightly higher LVEF, and lower NTpro-BNP values in comparison to patients with normal C3c values. No differences were found regarding NYHA functional class. Significantly more patients with elevated C3c had preexisting diabetes. The prevalence of CAD, arterial hypertension, and atrial fibrillation was not increased in patients with elevated C3c. *Conclusion.* Elevated C3c levels are associated with less adverse remodeling and improved survival in patients with stable systolic heart failure.

## 1. Introduction

Patients with heart failure frequently exhibit a chronic low-grade activation of the immune system as indicated by increased levels of cytokines, chemokines, and inflammatory proteins [1–3]. For many years it remained unclear how in heart failure, a primarily noninfectious disease with the rare exception of infectious myocarditis, the immune system could be triggered. However in recent years, it became clear that these reactions might be due to activation of the innate immune system via endogenous “danger signals” [4–6]. These danger signals are released, for example, by dying cells and include factors like heat shock proteins.

Effects of immune activation on heart failure development are time dependent. After an event of acute cardiac injury, like myocardial infarction, activation of the innate immune system is a prerequisite for adequate healing [7, 8]. However, long-term chronic innate immune activation is detrimental leading to adverse left ventricular remodeling and aggravation of heart failure [3, 4, 9].

Complement might be an important mediator in this context. The complement system is one of the key components of the innate immune system [10, 11]. It has a dual role: on the one hand, it is a receptor, for example, for host infection. On the other hand it is also an effector protein that can efficiently attract inflammatory cells and also directly destroy cells by the membrane attack complex. From noncardiac diseases we know that inappropriate complement activation is pathologic and leads to various autoimmune diseases [12, 13].

The complement system features more than 20 different serum proteins that are produced by a variety of cells. The interaction of these proteins constitutes a meticulously regulated cascade of activation steps. Finally, all activation steps converge on the complement factor C3. In the heart, activated C3 (C3a) caused tachycardia, impairment of atrioventricular conduction, left ventricular contractile failure, coronary vasoconstriction, and histamine release after injection into isolated guinea pig hearts [14]. Thus, C3 seems to be not only a good indicator for overall complement activation but might also be of pathophysiological relevance in the cardiovascular system. We therefore hypothesized that C3 would be a good marker of innate immune activation and might also be associated with adverse cardiac remodeling and mortality in patients with stable heart failure.

A problem with using complement components as biomarker is that due to their aggressive nature complement factors have a very short half-life. C3c is the stable conversion product of C3, which develops out of C3 within one hour at body temperature. For the detection of complement function, we used therefore C3c [15, 16], in a study cohort of patients with stable heart failure.

## 2. Methods

### 2.1. Patients

Patients participating in the Interdisciplinary Network for Heart Failure Study (INH Study, http://www.controlled-trials.com/, ISRCTN23325295) were eligible. The INH Study investigated the effects of a telephone-based nurse intervention on clinical outcome and enrolled consecutive adults hospitalized for congestive cardiac failure at nine hospitals in Bavaria and Baden-Württemberg. Inclusion criteria were informed written consent, left ventricular ejection fraction (LVEF) ≤40%, and typical signs and symptoms of heart failure at the time of inclusion. Exclusion criteria were logistic or health reasons precluding participation in a telephone-based intervention. Approval of the INH study protocol was obtained from the responsible Ethics Committees [17, 18].

### 2.2. Data Collection and Follow-Up

Prior to discharge, patients underwent a standardized evaluation including detailed medical history, physical examination including assessment of New York Heart Association (NYHA) functional class, blood chemistry, 12-lead electrocardiogram, and echocardiography. All assessments were repeated at 6-month intervals after hospital discharge. Survival status after 3 years was ascertained by contacting patients themselves or their general physician [17].

### 2.3. Laboratory Tests, C3c Sampling, and Measurement

Consecutive serum C3c levels of 197 patients were collected between January 2009 and June 2011 during an ambulatory outpatient visit, about 3 (1–7) years after the inclusion in the original study. Due to heart failure associated mortality and impossibility to attend the ambulatory follow-up, we could assess C3c values in 197 patients of the originally included 1022 patients. After rapid centrifugation of the venous blood sample collected between 8 and 11 AM, C3c was immediately measured in the Central Laboratory of the University Hospital Würzburg that accords with rigid external control. In clinical routine, complement C3c is measured instead of complement C3 [19–21] using turbidimetry (Cobas c 502, Roche, Mannheim, Germany). In our laboratory, the reference interval in adults spans from 75 to 140 mg/dL.

### 2.4. Definition of C3 Status

According to assay dependent reference intervals the patients were subdivided into three groups: low C3c (<75 mg/dL), normal C3c (75–140 mg/dL), and elevated C3c (>140 mg/dL). We evaluated the clinical characteristics, the survival, the echocardiographic status, and the laboratory parameters of the patients according to the predefined subgroups.

### 2.5. Data Analysis

Patients were grouped according to normal (75–140 mg/dL) versus elevated (>140 mg/dL) C3c levels; 33 (16.8%) of all evaluated heart failure patients had elevated C3c levels. Only one patient had a C3c level below the reference range and was excluded from the following analyses. Thus, C3c levels of 196 patients were included in the present analysis. Data are expressed as mean (standard deviation), median (quartiles), or *n* (%), as appropriate. Group-wise comparisons were performed using Fisher's exact test, chi-square test, Mann-Whitney *U*-test, or Kruskal-Wallis test, as appropriate. Kaplan-Meier curves were constructed to investigate the prognostic value of C3c and were tested by log rank test. Adjustment for potential confounders for the association between C3c and survival was performed using Cox proportional hazards regression, and hazard ratios (HR) with 95% confidence intervals (CI) were reported. Patients with normal C3c values served as reference. Reported *P* values are two-sided, and *P* values < 0.05 were considered statistically significant. All tests were performed using commercial software (SPSS Inc, Chicago, Illinois version 20.0).

## 3. Results

### 3.1. Study Subjects

A total of 196 patients with a median age of 69.0 years (range 32–87 years) were included in the analysis. No differences regarding age were found in patients with normal versus elevated C3c values (*P* = 0.77). Baseline characteristics of the entire sample are shown in Table 1. Forty-two (27%) of the patients were female, with a trend for a higher proportion of women in the group with elevated C3c (30% versus 20%, *P* = 0.174; Table 1). No differences were found regarding the prevalence of arterial hypertension (*P* = 0.636), coronary artery disease (*P* = 0.565), chronic obstructive pulmonary disease (*P* = 0.525), malignant disease (*P* = 0.523), and peripheral artery occlusive disease (*P* = 0.582) in patients with elevated C3c compared to those with normal C3c. Twenty-seven (82%) patients with elevated C3c had sinus rhythm and 5 (15%) atrial fibrillation on the actually performed ECG. The one remaining patient (3%) had a pacemaker dependent rhythm. A trend towards more frequent sinus rhythm (82% versus 66%; *P* = 0.079) was observed in patients with elevated C3c. No differences were observed regarding the frequency of atrial fibrillation (15% versus 24%; *P* = 0.272). Interestingly, the prevalence of diabetes mellitus was higher in patients with elevated C3c compared to those with normal C3c (61% versus 42%, *P* = 0.047). Patients with elevated C3c values had a slightly higher frequency of ICD and CRT device implantations at the C3c evaluation (27.3% versus 19.6% and 27.3% versus 11.0%). No differences in cardiac medication were found in patients with normal versus elevated C3c (Table 1).

### 3.2. Echocardiography

**Figure 1:**
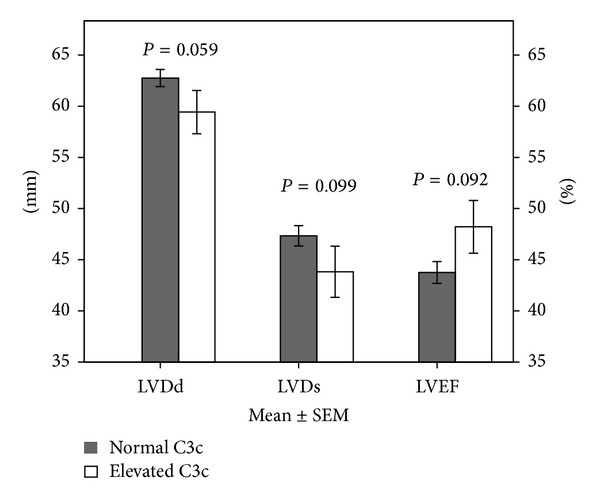
Comparison of echocardiographic parameters in patients with normal versus elevated C3c values. There is a trend towards smaller diastolic (59 ± 11 mm versus 63 ± 10 mm; *P* = 0.059) and systolic (44 ± 13 mm versus 47 ± 13 mm; *P* = 0.099) diameter of the left ventricle and higher left ventricular ejection fraction (48.0 ± 12.83% versus 43.5 ± 13.2%; *P* = 0.092) in patients with elevated complement C3c. LVDd, diastolic left ventricular diameter; LVDs, systolic left ventricular diameter; LVEF, left ventricular ejection fraction.

We found a trend towards smaller end-diastolic (59 ± 11 mm versus 63 ± 10 mm; *P* = 0.059) and end-systolic (44 ± 13 mm versus 47 ± 13 mm; *P* = 0.099) diameters of the left ventricle and towards a higher left ventricular ejection fraction (48.0 ± 12.83% versus 43.5 ± 13.2%; *P* = 0.092) in patients with elevated complement C3c (Figure 1). No relevant differences could be found regarding diastolic function (*P* > 0.05) and systolic tricuspid valve gradient (*P* > 0.05).

### 3.3. Laboratory Parameters

**Figure 2:**
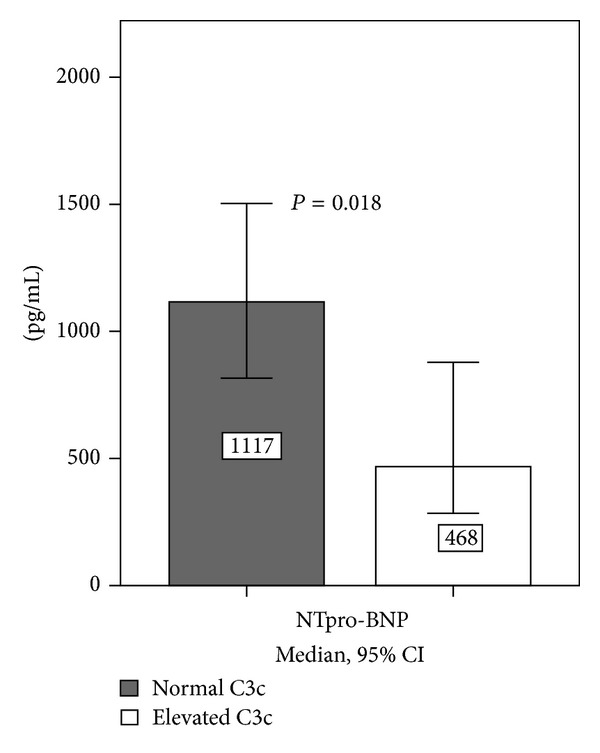
Comparison of NT-proBNP values in patients with normal versus elevated C3c values. Lower values of NTpro-BNP were found in patients with elevated C3c (468 [246; 1182] pg/mL versus 1117 [385; 2662] pg/mL; *P* = 0.018). NT-proBNP: N-terminal prohormone of brain natriuretic peptide.

Lower values of NTpro-BNP were found in patients with elevated C3c (468 [246; 1182] pg/mL versus 1117 [385; 2662] pg/mL; *P* = 0.018; Figure 2). C3c was negatively correlated with NTproBNP (*R* ^2^ = −0.266;*P* = 0.001). Atrial fibrillation is known to be associated with BNP levels and was underrepresented in patients with elevated C3c values. Nonetheless, two-way ANOVA did not show relevant interactions between C3c group allocation, occurrence of atrial fibrillation, and NTpro-BNP distribution.

C-reactive protein (CRP) values above 0.5 mg/dL (i.e., the upper threshold of normal range according to our Hospital Laboratory) were considered elevated. CRP levels were significantly higher in patients with elevated C3c (0.91 ± 0.18 mg/dL versus 0.55 ± 0.08 mg/dL; *P* < 0.001). We did not find any association between LV function or morphology in patients with normal versus elevated CRP (LV enddiastolic diameter: 62 ± 10 versus 62 ± 10 mm, *P* = 0.973; LV endsystolic diameter: 46 ± 12 versus 47 ± 12 mm, *P* = 0.770; LVEF: 45 ± 13 versus 44 ± 14%, *P* = 0.459). Kaplan Meier survival analysis showed a tendency for a better survival in patients with normal versus elevated CRP, independent of C3c status (Log rank 0.081).

We found relevant alterations in the blood profile of patients with elevated C3c: higher values for platelets (252 ± 108 versus 202 ± 53∗1000/*μ*L; *P* = 0.001), erythrocytes (4.73 ± 0.46 versus 4.50 ± 0.58∗10*E*6/*μ*L; *P* = 0.040), and leukocytes (8.45 ± 2.76 versus 7.45 ± 3.92∗1000/*μ*L; *P* = 0.016) but no differences regarding haemoglobin (13.94 ± 0.14 versus 14.14 ± 0.25 g/dL; *P* = 0.70). Ferritin levels were significantly higher (227 [152; 441] versus 161 [84; 325] *μ*g/L; *P* = 0.044) in patients with elevated C3c and were accompanied by corresponding alterations of transferrin saturation (22 ± 8 versus 26 ± 10%; *P* = 0.118). No differences were observed regarding the renal and liver function between the two groups.

### 3.4. Survival Analysis

**Figure 3:**
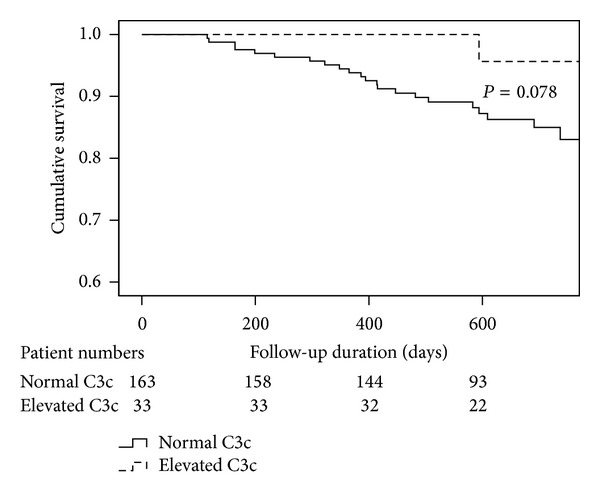
Kaplan-Meier estimates all-cause mortality risk by C3c values. Mortality risk tended to be increased in the group with normal (*n* = 163) C3c values compared to subjects with elevated (*n* = 33) C3c values (log rank test, *P* = 0.078).

During the follow-up period (median 21 months; range 3–43 months) 23 (11.7%) patients died. The follow-up duration for survivors comprised at least 9 months (median 22; range 9–43). Twenty-two patients (13.5%) in the group with normal C3c compared to only 1 (3.0%) patient in the group with elevated C3c died during the follow-up. Mortality risk tended to be increased in the group with normal C3c values compared to elevated C3c values (log rank 0.078; Figure 3). This corresponded to an unadjusted hazard ratio of 5.0 (95% CI 0.68–37.3, *P* = 0.114). This trend for an increased mortality risk in patients with normal C3c values was only slightly attenuated after adjustment for age (HR 4.13, 95% CI 0.55–30.8, *P* = 0.166) and sex (HR 3.98, 95% CI 0.52–30.8, *P* = 0.185) but notably attenuated after adjustment for NYHA functional class (HR 3.4, 95% CI 0.44–25.1, *P* = 0.242).

## 4. Discussion

Since there is good experimental evidence from studies in small animals that complement activation is mechanistically involved in adverse cardiac healing and remodeling after myocardial infarction [22], we hypothesized that complement plasma levels may be indicative for heart failure progression in humans. However, here we present data that elevated complement C3c levels in patients with heart failure are associated with a trend towards improved survival and better cardiac reverse remodeling (i.e., reduced left ventricular volume, increased ejection fraction, and reduced NT-proBNP values).

### 4.1. Complement—A Prognostic Marker in Heart Failure?

Several reports confirm that complement is activated after myocardial infarction [23–25]. Hill and Ward demonstrated C3 cleavage in the infarcted myocardium and documented a role for the complement system in leukocyte infiltration [26]. Moreover, complement inhibition consistently attenuated leukocyte recruitment following myocardial infarction highlighting the critical role of the complement cascade in triggering inflammation in the ischemic myocardium [25, 27]. Its role as a prognostic marker in heart failure was only addressed by a very limited number of investigations: Gombos et al. recently presented data from 182 patients indicating an association between activated complement C3a and a combined endpoint consisting of all-cause mortality or rehospitalization due to progression of heart failure [28]. Aukrust et al. found systemic complement activation in 39 patients with chronic heart failure; treatment with intravenous immunoglobulin reduced complement activation and increased left ventricular function during the 5-month follow-up period [29, 30]. However, both reports have major weaknesses: the patient cohort of Gombos et al. appears rather heterogeneous since the authors included not only outpatients but also inpatients after best possible cardiac recompensation. Moreover, variables allowing the assessment of left ventricular remodeling were not presented in this study. In contrast, our patients were all stable outpatients undergoing long-term follow-up in a clinical study with in-depth characterization of cardiac remodeling by echocardiography. The main limitation of the study by Aukrust et al. was the low number of patients studied. A further methodological problem is in both studies related to the complement measurements. Upon activation of complement factor C3, C3 is cleaved in two fragments: C3a and C3b. In the cited studies either C3a or C3b was measured. However, both components are unstable and are degraded upon freezing making a more or less immediate measurement necessary [16, 19–21]. In both studies samples were frozen prior to analysis, thus rendering the obtained complement plasma levels questionable. We circumvented this problem in two ways. First, we measured complement levels routinely without freezing our samples. Second, we used C3c, a stable conversion product which develops out of C3 within one hour at body temperature [15]. The disadvantage of C3c measurement is that we determined total C3 and cannot distinguish between activated and non-activated complement.

Our sample patients were clinically stable, under long-term heart failure medication and still under observation 3 to 4 years after the inclusion in the original study. So, one potential limitation of our study could be that our patients were not sick enough to develop immune activation. However, activation of an immune response in our cohort could be demonstrated by an association of C3c and increased leucocytes and C-reactive protein levels. Moreover, our study may be biased since our assessment was performed only once, while cardiac remodeling is an ongoing process. Ischemic cardiac disease is the predominant cause for heart failure in our patient cohort. For this reason, our data are underpowered to assess the role of C3c in patients with alternative causes of heart failure. Finally, natriuretic peptides might directly cause proinflammatory protein release [31–33]. Thus, one has to consider that inflammatory markers might not necessarily be independent markers of heart failure progression.

### 4.2. Are Peripheral Complement Levels a Marker of Intracardiac Complement Activation?

Heart failure is associated with activation of immune system [34]. Peripheral levels of important innate immune cytokines like tumor necrosis factor (TNF)-*α* are associated with adverse outcome [2, 3, 25, 35]. On the other side, infusion of TNF-*α* or intracardiac overexpression of TNF-*α* in animals leads to heart failure [36, 37]. This suggests that peripheral levels of TNF-*α* reflect a local, intracardiac activation of the immune system. However, this assumption cannot be generalized. For example, we know from our own studies that intracardiac levels of extracellular matrix proteins are not mirrored in the plasma [38]. In rheumatoid arthritis activation of complement by immune complexes in the joint space of patients results in local depression of C4 [39]. However, serum levels of C4, and more specifically C3, are elevated in serum of patients with rheumatoid arthritis [40, 41]. Thus, peripheral blood complement levels can only partly reflect complement involvement in the disease development and healing processes in the target tissue.

Moreover, in the clinical routine low complement levels are usually an indicator of high tissue complement turnover, for example, in glomerulonephritis [42]. Thus, higher peripheral levels of complement could also reflect a decreased activation of complement in the tissue. This could also explain why patients with higher peripheral—and potentially lower local—complement activation had a better clinical outcome in our study.

## 5. Conclusion

We present data that elevated C3c levels seem to be associated with less adverse remodeling and improved survival in patients with stable systolic heart failure. It is unlikely that plasma complement mirrors intracardiac complement activation.

## Figures and Tables

**Table 1:** Baseline characteristics of study participants.

	All patients^∞ ^(*n* = 197)	Normal C3c (*n* = 163)	Elevated C3c (*n* = 33)
Age (years)	67 (12)	68 (11)	63 (13)
Female sex	42 (21)	32 (20)	10 (30)
Duration of follow-up for survivors [months]	20 (6)	20 (6)	22 (4)
All-cause mortality	23 (12)	22 (14)	1 (3)*
Diagnosis of heart failure known			
≤5 years	77 (39.1)	63 (38.7)	14 (42.4)
5 years	103 (52.3)	87 (53.4)	15 (30.7)
Predominant cause of heart failure			
Coronary artery disease	92 (46.7)	78 (48)	13 (39.4)
Dilated cardiomyopathy	64 (32.5)	50 (31)	14 (42.4)
Hypertension	19 (9.6)	17 (10)	2 (6.1)
Other	22 (11.2)	18 (11)	4 (12.1)
NYHA functional class			
I/II	144 (73.1)	118 (72.4)	25 (75.8)
III/IV	53 (26.9)	45 (27.6)	8 (24.2)
Left ventricular ejection fraction (%)	44.2 (13.2)	43.5 (13.2)	48.0 (12.8)
Medical history			
Current smoker	16 (8.1)	14 (8.6)	2 (6.1)
Myocardial infarction	81 (41.1)	69 (42.3)	11 (33.3)
Comorbidities^†^			
Atrial fibrillation	44 (22.3)	39 (23.9)	5 (15.2)
Peripheral vascular disease	35 (17.8)	28 (17.2)	7 (21.2)
Hypertension	168 (85.3)	138 (84.7)	29 (87.9)
Diabetes mellitus	89 (45.2)	68 (41.7)	20 (60.6)*
COPD	31 (15.7)	27 (16.6)	4 (12.1)
Anemia	36 (18)	29 (18)	6 (18)
Renal dysfunction	96 (48.7)	78 (47.9)	17 (51.5)
Uncured malignancy	2 (1)	2 (1.2)	0
Devices			
ICD	41 (20.8)	32 (19.6)	9 (27.3)
CRT	27 (13.7)	18 (11.0)	9 (27.3)*
Medication			
ACEi and/or ARB	189 (96)	156 (96)	32 (97)
*β*-blocker	182 (92)	148 (91)	33 (100)
Aldosterone antagonist	108 (55)	87 (54)	21 (64)
Diuretic	170 (86)	139 (85)	30 (91)
Amiodarone	19 (9.6)	16 (9.8)	3 (9.1)
Digitalis	72 (37)	59 (36)	13 (39)

Values are mean (SD) or *n* (%). ^∞^All patients imply results for all patients with the C3c measurement, including the only one with the diminished C3c value. *P value < 0.05 in Mann-Whitney *U* test, the comparison between the group with normal C3c and elevated C3c. °Heart rate according to electrocardiogram.

^†^Comorbidities: atrial fibrillation: diagnosed from the electrocardiogram. Hypertension: sitting blood pressure > 140/90 mmHg or history of hypertension prior to the onset of heart failure or hypertensive heart disease accepted as predominant cause of heart failure. COPD: chronic obstructive pulmonary disease: history of this condition requiring bronchiolytic treatment or newly diagnosed according to the Global Initiative for Chronic Obstructive Lung Disease criteria [43]. Anemia according to WHO criteria: haemoglobin <12 g/dL in women and <13 g/dL in men [44]. Renal dysfunction: estimated glomerular filtration rate < 60 mL/min/1.73 m² [45]. ICD: implantable cardioverter-defibrillator. CRT: cardiac resynchronization therapy with a biventricular defibrillator; ACEi: angiotensin-converting enzyme inhibitor; ARB: angiotensin receptor blocker.
